# Infrastructure-Less Indoor Localization Using the Microphone, Magnetometer and Light Sensor of a Smartphone

**DOI:** 10.3390/s150820355

**Published:** 2015-08-18

**Authors:** Carlos E. Galván-Tejada, Juan Pablo García-Vázquez, Jorge I. Galván-Tejada, J. Rubén Delgado-Contreras, Ramon F. Brena

**Affiliations:** 1 Programa de Ingeniería de Software, Unidad Académica de Ingeniería Eléctrica, Universidad Autónoma de Zacatecas, Ciudad Universitaria Siglo XXI, Edificio de Ingeniería de Software e Ingeniería en Computación, Zacatecas 98160, Mexico; 2 School of Engineering, MyDCI, Autonomous University of Baja California (UABC), Mexicali 21100, Mexico; E-Mail: pablo.garcia@uabc.edu.mx; 3 Ingeniería Robótica y Mecatrónica, Unidad Académica de Ingeniería Eléctrica, Universidad Autónoma de Zacatecas “Francisco Garcia Salinas”, Zacatecas 98000, Mexico; E-Mail: gatejo@gmail.com; 4 Graduate School of Engineering and Science, Instituto Tecnológico de Monterrey, CETEC South, 5th Floor, Av. E. Garza Sada 2501, Monterrey, NL 64849, Mexico; E-Mails: ruben.dc@gmail.com (J.R.D.-C.); ramon.brena@itesm.mx (R.F.B.)

**Keywords:** indoor location, information fusion, feature extraction, feature selection, genetic algorithms

## Abstract

In this paper, we present the development of an infrastructure-less indoor location system (ILS), which relies on the use of a microphone, a magnetometer and a light sensor of a smartphone, all three of which are essentially passive sensors, relying on signals available practically in any building in the world, no matter how developed the region is. In our work, we merge the information from those sensors to estimate the user’s location in an indoor environment. A multivariate model is applied to find the user’s location, and we evaluate the quality of the resulting model in terms of sensitivity and specificity. Our experiments were carried out in an office environment during summer and winter, to take into account changes in light patterns, as well as changes in the Earth’s magnetic field irregularities. The experimental results clearly show the benefits of using the information fusion of multiple sensors when contrasted with the use of a single source of information.

## 1. Introduction

Determining the physical location of a user has become an important aspect of mobile computing, because the user’s location is a fundamental part of her/his contextual information, allowing applications to be better suited to the user’s situation [1]. Beyond the widespread use of GPS location systems, which have been incredibly useful outdoors, indoor location has been proven to be a different problem, both because the buildings partially block the GPS signal, thus reducing precision, and, also, because of a higher concentration of relevant places, so the precision needed indoors is much higher. Thus, in recent years, the development of indoor location systems (ILS) has been under constant improvement, especially with the availability of new small and inexpensive sensors. There are several technological approaches that have been proposed for the design of indoor location systems. These include infrared light (IR), ultrasonic sensors, wireless local area networks (WLAN), radio frequency identification (RFID), Bluetooth, ultra wideband (UWB), ZigBee and computer vision, among others [2,3,4]. The combination of these technologies has also been considered [5,6]. Based on these technologies, various ILS have been developed, such as active badge [7], active bat [8], cricket [9], LANDMARC [10], Bluepos [11], LOSNUS [12], CLIPS [13], *etc.* However, most of them cannot be deployed in a mobile phone and present the disadvantage of requiring a dedicated infrastructure, hindering the system’s scalability as it requires adding devices, and in some cases, when an ILS is based on computer vision algorithms, high processing capabilities are required.

Therefore, some modern ILS are based on the use of a variety of sensors and devices that are embedded in smartphones (e.g., accelerometer, gyroscope, magnetometer) [14,15]; these can be classified as follows:

Inertial-based mobile ILS: these are based on the inertial sensors of a smartphone (e.g., accelerometers and gyroscope). The accelerometer can be used to determine the changes in the user’s position produced when an acceleration is detected in one or more axes, while the gyroscope can be used to detect the changes in the direction to improve the location estimation. Examples of these type of systems are presented in Li *et al.* [16] and Pratama *et al.* [17]. The main disadvantage of these systems is that several issues must be considered in the model for estimating the user’s location correctly, such as: (i) accurate knowledge of the initial reference point; (ii) the changes in the position and orientation of the device to the user’s body; and (iii) a calibration phase for estimating the stride length of the user that will use the system.

Camera-based mobile ILS: In these systems, the camera of the smartphone is used to capture information from the user’s location (e.g., an image, video, markers or codes). This information is then compared to the reference information that was previously collected. Examples of this type of system are MoVIPS [18] and SIngPost [19]. The main disadvantage of this approach is that it requires high processing capacity, and the accuracy of the system is reduced when the quality of the information captured presents low resolution or motion blur.

Signal-based mobile ILS: These systems use the sensors embedded in a smartphone to detect, measure and capture signals that are emitted by other devices and then conveyed inside the indoor environment (e.g., Wi-Fi, Bluetooth), as well as the natural signals that are commonly found in those indoor environments (e.g., magnetic field, ambient sound). In these systems, the position estimation is commonly performed through methods, such as “fingerprinting”. This one is composed of two phases: training and position determination. Firstly, a map of the observed signal strength values measured at different locations is recorded during a training phase. Secondly, the signal strength values observed at a user’s device are compared to the map values by using proximity matching algorithms, including, but not limited to k-NN (k-nearest neighbors) [10]. Examples of these systems are presented in Storms *et al*. [20] and Bilke *et al.* [21]. The main disadvantage is that they involve the previous point-by-point mapping of a given indoor environment, measuring the magnitude and/or direction of a specific signal at each point and, then, using this signal map for location purposes, finding the most similar place in the signal map to the one detected at a given point.

In this paper, we propose a signal-based ILS, which relies on merging the information from a microphone, a magnetometer and a light sensor embedded in a smartphone to estimate the user’s location in an indoor environment. The goal of our approach, however, is not to find a point in a coordinate system for the user’s location, but just a “room” or office in which the user is. For most practical purposes, this room-level location is large enough; this perspective has also the implication that in order to measure the location quality, instead of expressing an average error distance, we just compare the room predicted by the system against the actual room, and the proportion of correct guesses is expressed in terms of sensitivity and specificity.

There is a similar project called SurroundSense [22], which also targets room-level localization with multi-modal sensor information. However, we identify two basic differences: (i) SurroundSense does not consider magnetic fields as an information source; and (ii) the information fusion technique proposed in SurroundSense sequentially filtered information sources using sound first, then movement and then color, while our methodology proposes to perform information fusion using all information sources at once, combining the different features to make the classification of places.

Furthermore, we have two important goals for our approach: one is to provide low computational cost methods that could be amenable to run on portable devices, and the other is to rely on signals that are available in practically any building in the world (as are ambient noise, ambient light and the magnetic field of the Earth), regardless of how developed the country in which the user lives is.

In order to perform the signal analysis for noise, luminosity and magnetic field, we use our methodology previously presented in Galván-Tejada *et al.* [23]. In our approach, there is no need to construct a detailed signal map, consisting of a grid of signal measurements for each point of the indoor environment, as performed by Storms *et al.* [20] and Bilke *et al.* [21]. Instead, we propose to store a “signature” taken from a random walk inside a given room. By “walk” we refer to walking with a non-predefined pattern, as opposed to a perimeter walk proposed in other approaches. To acquire the information necessary for applying the model proposed in our methodology, we consider the temporal and spectral representation of the evolution of the magnetic field, the environmental sound and the indoor light signals. This method has been shown to be independent of the exact path of the user at the time of signal acquisition, thus facilitating the data acquisition phase and eliminating the necessity of creating an environmental map construction.

The main contribution of this paper is the fusion strategy to merge the information signals necessary to estimate the user’s location in an indoor environment. These signals include the magnetic field of the Earth, the environmental background sound and the luminosity pattern at each building’s room or office, which are present in any indoor environment. This is the rationale for considering the combination of these three signals for building an ILS that could be applied in a wide variety of indoor environments.

This paper is organized as follows. Our proposed method for estimating the user location based on merging data from the magnetic field, environmental audio and indoor light intensity is described in Section 2. In Section 3, we present the experiments and results. A discussion of our results is presented in Section 4, and finally, our conclusions and future work are presented in Section 5.

## 2. Indoor Location Estimation Methodology

**Figure 1:**
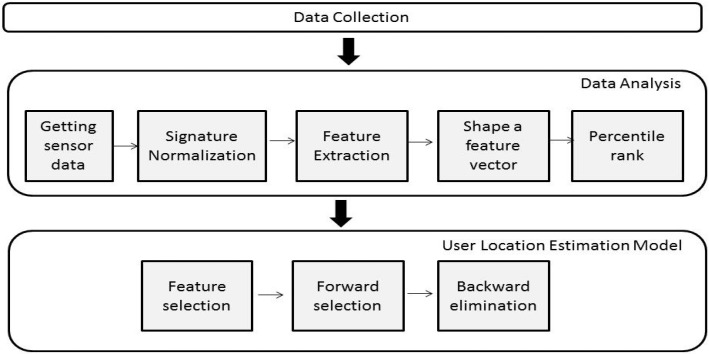
Methodology for estimating the user location.

For estimating the user’s location in an indoor environment, we extend our previous indoor location methodology presented in Galván-Tejada *et al.* [23]. This extension consists of the incorporation of two information sources (light and sound; the previous work was only about magnetic fields) and the corresponding adjustment of the multivariate methodology. Additionally, because increasing the number of information sources implies more features, we add two steps in the last phase (user location estimation model), a forward selection and a backward elimination step, which allow us to reduce features from the estimation model. This extended methodology consists of three phases, as is shown in Figure 1. The three phases are described in the following.

### 2.1. Data Acquisition

This phase consists of acquiring information of the Earth’s magnetic field irregularities, environmental audio and indoor light intensity information from an indoor environment. To acquire this information, we developed a smartphone application that can access data from the microphone, the magnetometer and the light sensor embedded in the smartphone. For getting data entries, the user must walk around with the smartphone carried on the user’s hand with the screen up, keeping it at the waist level. This activity must be carried out during 10 seconds with an approximate speed of 1 m/s (we have verified that the exact speed is immaterial). In our proposal, the user’s location can be estimated regardless of the walking pattern, as proposed by Gozick [24].

The data are then used to form signatures, *i.e.*, a basic set of data entries that represent the spectral and temporal behavior of the signals captured in the room. To estimate the number of signatures needed to create a model, we use Equation (1) proposed by Eberhardt [25]. It allows us to determine the minimal number of experiments in a multivariate process having statistical validity. In Equation (1), *x* is the minimum number of experiments and *N* is the number of rooms multiplied by the number of features and the number of signals, where the features are obtained from the captured signals. A detailed description of the features considered is provided later in the document.

(1) $$x=log_{2}\left(N\right)+1$$

### 2.2. Data Analysis

This phase consists of five tasks, as shown in Figure 1, which are described as follows.

Getting sensors Data: The information provided by the sensors can be represented in vector form. For all of the sensors, 100 readings per second were recorded; the light sensor gets the quantity of lumens represented by a scalar; in the case of the audio sensor, the amplitude of the audio wave is stored. However, with regard to the magnetic field data vector, we need to compute the magnitude of the magnetic field at each measurement point. Each magnetic field measurement is composed of three elements: $B_{x},B_{y}$ and $B_{z}$, and the magnitude of the magnetic field is computed by Equation (2), where $M_{x},M_{y}$ and $M_{z}$ (*M* referring to the local or current location of the magnetic field) are the three physical axes along x,y and *z*, respectively.

(2) $$\left|M\right|=\sqrt{M_{x}^{2}+M_{y}^{2}+M_{z}^{2}}$$

Signature normalization: After all sensor measurements were obtained, the resulting vector data are processed to eliminate spatial scaling and shifting. This is accomplished by normalizing each signature using Equation (3), where $z_{i,d}$ is the normalized reading, $r_{i,d}$ refers to the *i*-th observation of the signature in dimension *d*, where *d* represents our data sources; $\mu_{d}$ is the mean value of the signature for dimension *d*, and $\sigma_{d}$ is the standard deviation of the signature for dimension *d*.

(3) $$\forall i\in m:z_{i,d}=\frac{r_{i,d}-\mu_{d}}{\sigma_{d}}$$

Equation (3) is applied for all dimensions in $R^{d}$.

Feature extraction: This process consists of extracting the minimal number of signal features that enable us to characterize the behavior of the signal. To select the appropriate set of features, we carried out a review of the literature in digital signal processing (DSP) and statistical analysis [26,27,28,29], and as a result, we identified 46 features shown in Table 1. From the features considered, 16 correspond to the temporal domain and 30 to the spectral domain. Temporal features are computed from the waveform of magnetic field, sound and light signals, while spectral features are acquired performing a P-point fast Fourier transform to each signature of the signals [20]. For computing the spectral features, we apply Equation (4), where $ES_{i}$ is the *i*-th energy signature of the normalized signal and $NS_{i}$ is the *i*-th normalized signature.

(4) $$\forall i\in n:ES_{i}=FFT\left(NS_{i}\right)$$

**Table 1:** Features extracted.

Features	Temporal Domain	Spectral Domain
Kurtosis	*	*
Mean	*	*
Median	*	*
Standard Deviation	*	*
Variance	*	*
Coefficient of Variation (CV)	*	*
Inverse CV	*	*
1,5,25,50,75,95,99 100-Quantile	*	*
Trimmed Mean	*	*
Shannon Entropy		*
Slope		*
Spectral Flatness		*
Spectral Centroid		*
Skewness		*
1–10 Spectrum Components		*

Conform a feature vector: Once all of the features are computed, all of them are merged into a set of features that summarizes the behavior of the signal. This reduces the amount of data, from 1000 data points to 46 features per signature.

Percentile rank: To keep each feature value in the range from 0 to 1, we get the percentile rank, using Equation (5), where *x* is the feature vector and *N* is the number of features. Note that Tasks 2 and 5 are necessary to keep all of the feature values in the same range and with the same weight for the feature selection process explained below in this document.

(5) $$PR=\frac{truncation(rank(x))}{\left(N+1\right)}$$

### 2.3. User’s Location Estimation Model

Three tasks must be performed to develop a model for estimating the user’s location; in each task, a model is acquired. The fitness of the models of the first two steps is calculated using the cross-validation approach and in the final task, which is the final model; the fitness is calculated using the split dataset approach, using 30 percent of the dataset as a blind test; these tasks are described as follows:

Feature selection (FS): The aim of this task is to reduce the number of features and to increase the accuracy of the model when less features are considered, thus avoiding the curse of dimensionality [30] and obtaining a less costly computational model.

A feature selection process can be viewed as an optimization problem. In this particular case, the fitness of the model must be optimized with the minimum number of features, and a well-know method for solving this kind of problem is the genetic algorithms (GA). There are other feature extraction techniques, like principal component analysis (PCA) and singular value decomposition (SVD). However, they discard some features that can be useful for the development of a location system based on using our methodology.

In feature selection, a genetic algorithm perform an analysis following 3 basic steps:

- From a random selection of subsets from a population, the chromosomes are defined as variable subsets of a given size.
- The capability of each chromosome is assessed for its ability to predict a dependent variable and has a certain level of accuracy.
- The natural selection process, progressive improvement of the chromosome population, is driven by a number of operators: selection, mutation and crossover.

We propose to perform the analysis of 300 chromosomes, which represents the classification models, of 5 genes (features) each, 200 generations in each model, using in this evolutionary cycle the nearest centroid classifier. These numbers or generations were chosen to cover a big number of combinations of features, and the chromosome size was selected to minimize the “curse of dimensionality”. A well-known feature selection strategy, 3 k-fold cross-validation, was used in the GA setup due to the number of available samples. This 3 k-fold step allows one to generate a rank of features, which depend on the capability of each feature to estimate the location.

Forward selection (FS): This task generates nested models using the rank of features, adding the next best-ranked feature, one at a time in an iterative process, so that it selects the features that increase fitness the most [31].

Backward elimination (BE): To remove possible redundant information in the model acquired with the FS strategy, a BE strategy is applied. This BE involves the testing and elimination of features from the FS model; when the deletion of one feature from the FS model improves the fitness of the model, it is removed; this process is repeated until no improvement is detected. The model after BE gets a better fitness than the others obtained using the GA and the FS strategy.

## 3. Experiments and Results

In this section, we describe first the experimental results obtained from each sensor separately, in order to acquire an individual model, and then, we present the procedure for making the fusion of all of the sensors’ information. This procedure is carried out for two different datasets, one collected for summer afternoons and another one for a winter morning.

### 3.1. Local Test Environment

The experiments were carried out in a multi-floor building, which we see as a typical indoor office environment. The dimensions of the floor are 11 m wide and 20 m long, with an area of 220 m${}^{2}$. This floor is composed of 20 offices, 2 meeting rooms and 4 corridors. The floor has 38 linear fluorescent lights (General Electric: F96T8-SPX41), 38 air conditioning ducts (which have an impact on the environmental noise) and one wall of the building composed entirely of windows, which affects mainly ambient light.

### 3.2. Test Data Collection

**Figure 2:**
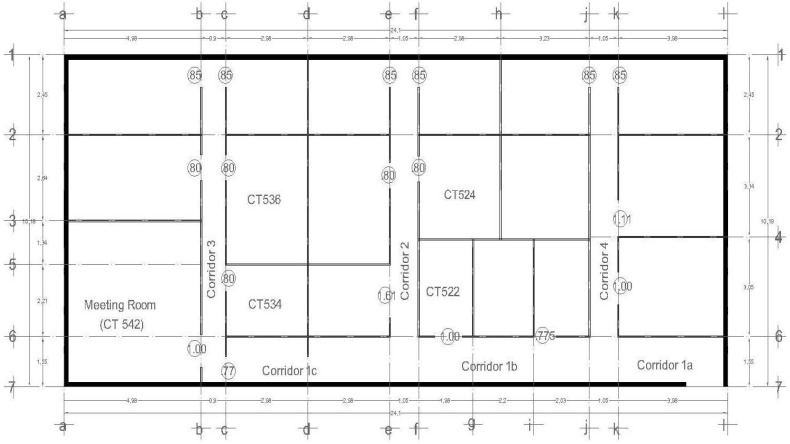
Floor layout.

The data collection took place in two different seasons and different times of day; one collection took place during summer in the afternoon (summer dataset) and the second one in winter during the morning (winter dataset) (data sets are available at: http://aaami.mty.itesm.mx/?page_id=24). Both were performed in 5 offices, 6 corridors and 1 meeting room. These spaces were randomly selected in order to cover several places around the floor; these offices and corridors are labeled in Figure 2.

To collect the data from the microphone, magnetic field (device model: YAS532) and light (device model: CM3323-RGB) sensors, we developed a mobile application in Java using the Google API Level 7. The application was executed in a smartphone Samsung Galaxy S4 i905 with Samsung official Android Version 4.1 rom (Jelly Bean) (data sets are available at: http://www.gsmarena.com/samsung_i9505_galaxy_s4-5371.php).

### 3.3. Software Requirements

To execute the proposed methodology, two software tools were required: (1) R project (data sets are available at: http://www.r-project.org/), a free software (GNU project) environment for statistical computing, multi-platform, which allowed us to manage the data because of its integrated collection of data analysis tools; and (2) Galgo, an R package based on a genetic algorithm variable selection strategy, primarily designed to develop statistical models from large-scale datasets [32]. The Galgo analysis protocol is composed of 4 steps:

Setting-up the analysis: The analysis using Galgo starts pre-processing the data where the user specifies classes, variables and GA parameters in order to comply with the requirements of Galgo. All of this process can be done using another R tool. Finally, the parameters that define the GA search environment are defined. For this experiment, we define as classes the room names, that is CT542, CT536, CT534, CT522, CT524, Corridor 1a, 1b, 1c, Corridor 2, Corridor 3 and Corridor 4. The variables were the features extracted (Table 1) from the signatures, and finally, 200 generations and a size of 5 genes for 300 chromosomes were defined.

Searching relevant multivariate models: An evolutionary cycle begins from a random population of chromosomes of a size predefined in the fist step, in the parameters of the GA. In this case, we start 300 evolutionary cycles with the same configuration to expand the number of combinations even more.

Refinement and analysis of the population for selected chromosomes: The GA procedure selects the chromosomes that have the desired classification accuracy. In this step, after the selection, an analysis of the genes can be done to reduce the possibility of having genes that do not contribute to the fitness of the model.

Selection of a representative statistical model: During all of the Galgo process, several models are generated, and in the final step, the best model is chosen.

### 3.4. Getting the Classification Models from the Information Sources

**Figure 3:**
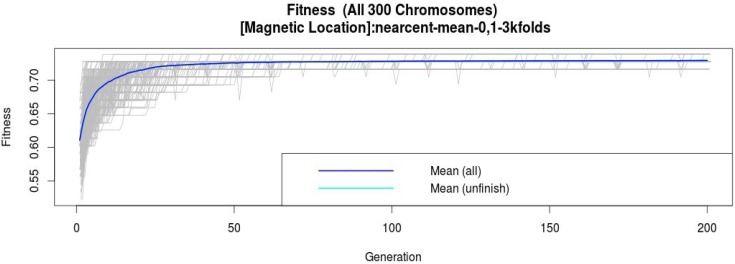
Magnetic field summer dataset evolution of the 300 nearest centroid models throughout 200 generations.

To obtain the best classification model of each sensor, an evolution of the 300 models during the GA process using the nearest centroid classifiers was done per sensor (including the microphone). The nearest centroid was chosen because it has been identified that this is a fast and simple algorithm for classifier, which in addition has the capability to overcome the incompleteness of some datasets [31]. For instance, Figure 3 shows the fitness evolution of these 300 classifiers from the summer magnetic field dataset. We can see how until the GA process is completed, the fitness increases because of the selection of the best features. The gray lines show the evolution of each of the models generated during this process; the mean of the fitness of all of the models is represented by a blue line, and the light blue line represents the mean of the fitness until the evolution is carried out.

**Figure 4:**
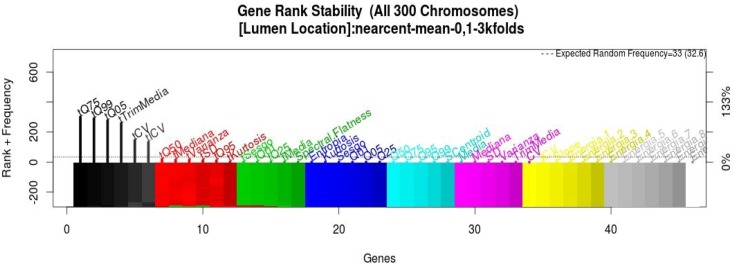
Light sensor summer dataset gene stability.

To estimate the accuracy of the models from the selected chromosomes, a cross-validation strategy was applied. This process is important, because during this, the 300 models generating all of the variables are ranked by frequency; this ranking helps to know which variables summarize the behavior with high accuracy. For example, in Figure 4 is shown the rank of the variables from the light sensor from the summer dataset. This ranking allows us to know the stability of the genes and to use this rank to obtain a new model following the forward selection strategy. We can see that in both cases, the features in the first places of the rank are from the temporal evolution.

Once the forward selection strategy is applied, the following models were obtained:

Magnetic field sensor (summer dataset): 1 model composed of 9 features selected from the top 30 ranked features.

Light sensor (summer dataset): 6 models composed of 7 to 17 features selected from the top 30 ranked features.

Microphone device (summer dataset): 5 models composed of 5 to 18 features selected from the top 30 ranked features.

Magnetic field sensor (winter dataset): 2 models composed of 9 and 10 features selected from the top 30 ranked features.

Light sensor (winter dataset): 6 models composed of 14 to 21 features selected from the top 30 ranked features.

Microphone device (winter dataset): 4 models composed of 7 to 10 features selected from the top 30 ranked features.

The final classification models for the sensors were obtained after applying a backward elimination strategy in order to avoid redundant information on the forward selection models. This feature elimination was applied to the models obtained with the forward selection strategy. The backward elimination strategy was carried out until no feature removal improves the fitness of the parent model. The cross-validation strategy was used to validate the final classification model, then the sensitivity and specificity were obtained for each sensor.

After the backward elimination, the classifier models obtained were the following:

Magnetic field sensor (summer dataset): a model composed of 8 features, 100-quantile 99, 95, 75 and 5, variance and media from the temporal signal and Component Number 2 and 100-quantile 5 from the spectral evolution.

Light sensor (summer dataset): a model composed of 6 features, all of them from the temporal evolution, 100-quantile 5, 50, 75 and 99, 90 percent trimmed media and the inverse coefficient of variation.

Microphone device (summer dataset): a model composed of 5 features, 100-quantile 5, 25 and variance from the temporal and Components 2 and 5 from the spectral evolution, equal to the best model obtained with the forward selection strategy.

Magnetic field sensor (winter dataset): a model composed of 9 features, standard deviation, media and 100-quantile 99, 95, 5 and 1, variance, coefficient of variation, inverse coefficient of variation and media from the temporal signal and spectral flatness from the spectral evolution.

Light sensor (winter dataset): a model composed of 9 features, 100-quantile 50, 75 and 95, 90 percent trimmed media, media, standard deviation and variance of the temporal evolution and Component Number 7 and 100-quantile 95 from the spectral evolution

Microphone device (winter dataset): a model composed of 6 variables, 100-quantile 5, 75 and 95 from the temporal and 100-quantile 75, 95 and 99 from the spectral evolution.

In Tables Table 2 and Table 3, we can see that even when the season is different, the sensitivity and the specificity of all of the sensors are similar. For example, the sensitivity of all of the sensors differs in an interval from 1 to 4 percent.

**Table 2:** Comparison of sensitivity and specificity: summer dataset.

Sensor/Device	Sensitivity	Specificity
Magnetic Field Sensor	0.7246683	0.9704668
Light Sensor	0.7059034	0.9705903
Microphone Device	0.7567806	0.9756781

**Table 3:** Comparison of sensitivity and specificity: winter dataset.

Sensor/Device	Sensitivity	Specificity
Magnetic- Field Sensor	0.7580685	0.9758069
Light Sensor	0.7030924	0.9703092
Microphone Device	0.776298	0.9776298

### 3.5. Signal Information Fusion

**Figure 5:**
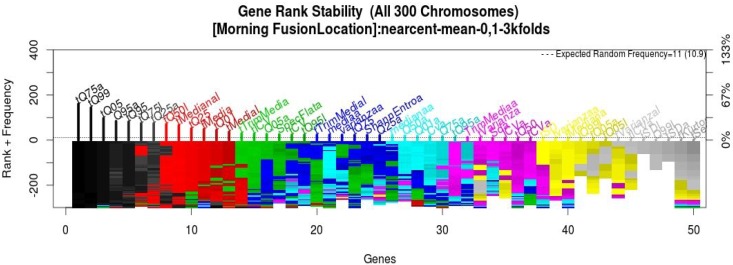
Winter dataset gene stability from the fusion of all sensors.

To merge the information from the sensors, the features from all of the sensors were merged into one dataset; this procedure was done for the summer and winter datasets, 138 features per room. The same methodology of Section 2 was applied to reduce the 138 features. From the GA process, the selection of the best chromosomes is done in order to rank the variables by frequency; this rank is generated with more features because it contains the variables from the sensors. For instance, the final rank for winter dataset is shown in Figure 5 to know the stability of the genes associated with features from sensors; this rank was used to obtain a new model following the forward selection strategy.

Once the forward selection strategy is applied, 24 models were obtained, 8 for the summer season and 16 for the winter season, composed of 6 to 50 features. As the fusion dataset has 138 features, the forward selection was applied from the top 50 ranked features instead of the first 30, as in the individual sources of information.

The final classification model of the fusion for each dataset was obtained after applying the backward elimination strategy.

After the backward elimination, the classifier model obtained for the summer dataset is composed of 7 features, 3 from the magnetic field sensor (100-quantile 99 and 75 from the temporal evolution and Component 1 from the spectral evolution), 3 from the microphone (100-quantile 99 and 5 from the temporal evolution and the kurtosis from the spectral evolution) and, finally, 1 from the light sensor (spectral flatness from the spectral evolution).

The model acquired from the backward elimination for the winter dataset is composed of 5 variables, 2 from the magnetic field sensor (100-quantile 5 and 95 from the temporal evolution), 2 from the microphone (100-quantile 75 from the temporal evolution and 100-quantile 95 from the spectral evolution) and 1 from the light sensor (100-quantile 75 from the temporal evolution).

**Figure 6:**
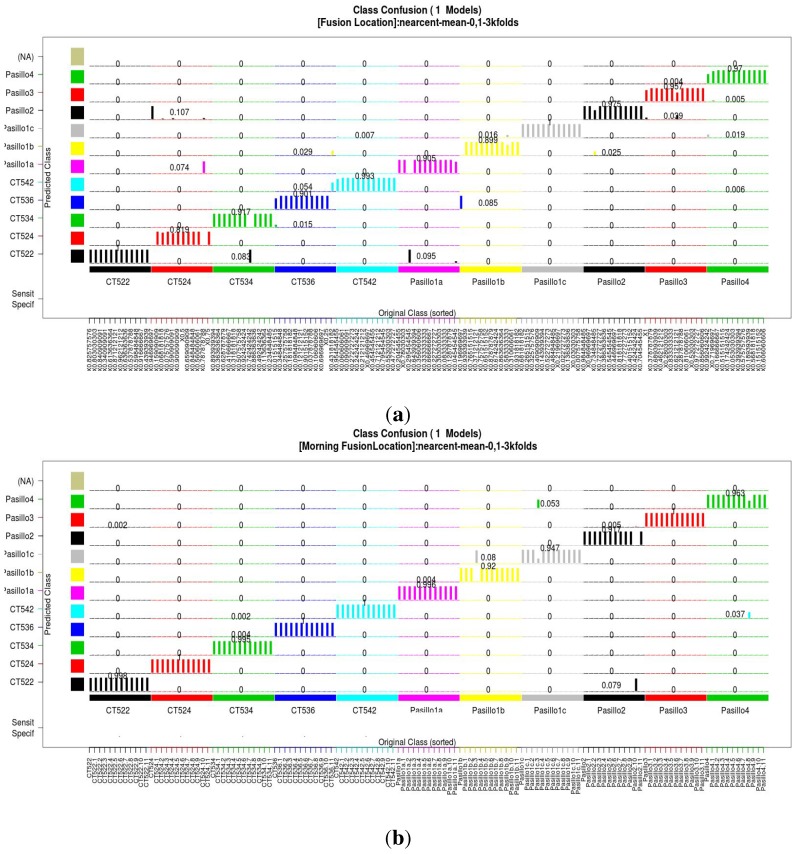
Confusion matrix plot acquired from the classification models. (**a**) summer dataset; (**b**) winter dataset.

The confusion matrix plot acquired from the classification models is shown in Figure 6; the specificity and sensitivity obtained from these two models merging information from all of the sensors is presented in Table 4, showing that the resulting model is better than the models obtained from the sensors individually, which are presented in Tables Table 2 and Table 3.

In order to evaluate the quality of the model acquired, we compare it against other commonly-used classification algorithms, able to deal with nonlinear relations, insensitive to missing values and capable of handling numerical and categorical inputs. The comparison is in terms of sensitivity; the results are presented in Table 5. Our approach outperforms all of them in two principal senses: (i) the number of features was evaluated given the importance in the computational cost; in this sense only the best chromosome requires a lower number of features than the final model acquired using our proposal; and (ii) the sensitivity was evaluated because of the potential to describe the behavior of the model. In this evaluation, our model outperforms all of the other methods. Our proposal allows one to fuse several information sources and to select only the most representative features, improving the fitness and reducing the amount of information needed from each source.

**Table 4:** Sensitivity and specificity from the final models.

Season Dataset	Sensitivity	Specificity
Summer Dataset	0.9396806	0.9939681
Winter Dataset	0.9760147	0.9976015

**Table 5:** Comparison of different approaches.

Approach	Features	Sensitivity
Best Chromosome	5	0.889
Nearest Centroid	136	0.920
Maximum Likelihood Classification	136	0.926
K-Nearest Neighbors	136	0.931
Random Forest	136	0.934
Our Approach	6	0.955

## 4. Discussion

In this research, we focus on using multivariate models as an implicit form of information fusion for taking into account features of the considered signals (magnetic field, environmental sound, light intensity) for indoor location. The results of the experiments presented in Section 3 enable us to establish that merging multiple sources of information to estimate the user location improves the sensitivity around 22% and specificity around 2% of the system in comparison to only using one source of information individually (e.g., magnetic field). In addition, that data fusion enables us to generate models with less signal features, as presented in Section 3.5, as we have reduced the number of signal features needed to generate an indoor location estimation model 94% for the summer model and 96% for the winter model, which has the consequence of reducing the computational cost of the location process (remember that one of our goals is to allow the location process to run on a smartphone).

Regarding the information sources, we notice that the surviving features are independent of time, because all of the surviving features require ordered data (100-quantile). Then, temporal shape features provide more information to locate a user in indoor environments.

An interesting issue regarding information sources was that the magnetic field signal source has more weight in our model to estimate the user location. This is due to the fact that magnetic fields do not have variations as other sources, such as indoor light intensity or environmental audio. The stability of the magnetic field signal for indoor location also has been reported in the ILS projects of Storms *et al.* [20] and Chung *et al.* [33].

In our project, we propose using the fingerprint method to generate a model for merging different sources of environmental information to locate people indoors.

Finally, our results indicate that the time of day or season does not affect our location model very much. This is because most of the features in the final models are from the magnetic field, and the stability of the signal is for extended periods of time.

## 5. Conclusions and Future Work

We have presented a methodology for estimating the location of a user carrying a portable device that comprises a magnetometer, a microphone and a luminosity sensor in an indoor environment.

Our method involves first the calculation of a set of features of the considered signals, including time features, spectral features and energy features; then, a feature selection process follows, involving the use of genetic algorithms, complemented with forward selection and backward elimination, giving a very reduced set of features. We have experimentally shown that this reduced set of features gives a very high precision for discerning the user location, in spite of the great information reduction achieved. Thus, we have provided evidence that multivariate models can be used as an information fusion technique, in order to estimate the user location in indoor environments.

The main contribution of this paper is the use of multivariate models as an implicit form of information fusion for taking into account features of the considered signals (magnetic, sound, luminosity) for indoor location, achieving the information reduction. Further, we have shown that incorporating several signals has indeed resulted in an improvement in the precision, which is of course a characteristic of good information fusion.

The signals that we selected (magnetism, sound and luminosity), which are strictly passive signals that do not require any infrastructure installation and are available in practically every single building in the world, make this method very widely applicable for indoor location.

As future work, we consider that this methodology is amenable to be applied to other combinations of input signals for indoor location when available, as for instance the wireless Internet access points’ signal intensity, Bluetooth and other signals that are already available in many current portable devices, so we intend to further investigate their use. We are also considering using crowdsourcing, a new paradigm that leverages ubiquitous mobile sensing devices for collaborative tasks [34], in order to avoid explicitly performing the calibration task for each room. This could enable us to automatically construct the magnetic map. In addition, we consider carrying out a survey about how many features can affect our system in different buildings and how you could take them into account to plan the data collection stage.
